# Enhancing Dendritic Cell Therapy in Solid Tumors with Immunomodulating Conventional Treatment

**DOI:** 10.1016/j.omto.2019.03.007

**Published:** 2019-03-27

**Authors:** Robert A. Belderbos, Joachim G.J.V. Aerts, Heleen Vroman

**Affiliations:** 1 Department of Pulmonary Medicine, Erasmus MC Rotterdam, the Netherlands; 2 Erasmus MC Cancer Institute, Erasmus MC Rotterdam, the Netherlands

**Keywords:** dendritic cell-based therapy, chemotherapy, radiotherapy, checkpoint inhibitors, immunotherapy, tumor microenvironment, regulatory T cells, myeloid-derived suppressor cells, immunogenic cell death, macrophages

## Abstract

Dendritic cells (DCs) are the most potent antigen-presenting cells and are the key initiator of tumor-specific immune responses. These characteristics are exploited by DC therapy, where DCs are *ex vivo* loaded with tumor-associated antigens (TAAs) and used to induce tumor-specific immune responses. Unfortunately, clinical responses remain limited to a proportion of the patients. Tumor characteristics and the immunosuppressive tumor microenvironment (TME) of the tumor are likely hampering efficacy of DC therapy. Therefore, reducing the immunosuppressive TME by combining DC therapy with other treatments could be a promising strategy. Initially, conventional cancer therapies, such as chemotherapy and radiotherapy, were thought to specifically target cancerous cells. Recent insights indicate that these therapies additionally augment tumor immunity by targeting immunosuppressive cell subsets in the TME, inducing immunogenic cell death (ICD), or blocking inhibitory molecules. Therefore, combining DC therapy with registered therapies such as chemotherapy, radiotherapy, or checkpoint inhibitors could be a promising treatment strategy to improve the efficacy of DC therapy. In this review, we evaluate various clinical applicable combination strategies to improve the efficacy of DC therapy.

## Main Text

Dendritic cells (DCs) are professional antigen-presenting cells (APCs) capable of inducing a potent immune response through the presentation of exogenous antigens.^1^ Immature DCs, efficient at engulfing and processing antigens, reside in the periphery, and they mature upon encounter with danger-associated molecular patterns (DAMPs) and pattern-associated molecular patterns (PAMPs).^2^ Upon encounter with these danger signals, DCs upregulate co-stimulatory molecules (CD80, CD86, and CD40) and chemokine receptors (e.g., CCR7), produce pro-inflammatory cytokines, and migrate to the lymph node to activate T cells.^3^ T cell activation is induced by antigen presentation (signal 1), co-stimulation (signal 2), and the secretion of pro-inflammatory cytokines (signal 3).^4^ In contrast, antigen presentation in the absence of signals 2 and 3 induces tolerance.^5^, ^6^ In a tumor setting, both tumor cells and immunosuppressive cells in the tumor microenvironment (TME) can hamper anti-tumor immune responses.^7^

In DC therapy production, DCs are loaded and matured *ex vivo* to circumvent the initial immunosuppressive influence of the TME and tumor cells on endogenous DC maturation. In addition, the administration of autologous DCs could induce and improve *in vivo* tumor-specific immune response. It is believed that DC therapy has not yet reached its full potential.^8^, ^9^, ^10^ The rather limited clinical efficacy of DC therapy can be dependent on DC therapy-related aspects, such as the choice of antigen, method of loading, or type of DCs used. Next to that, active immunosuppression by the tumor and the TME could also hamper the immune-activating potential of the administered DCs and suppress the function and infiltration of activated T cells.^11^, ^12^, ^13^

**Figure 1:**
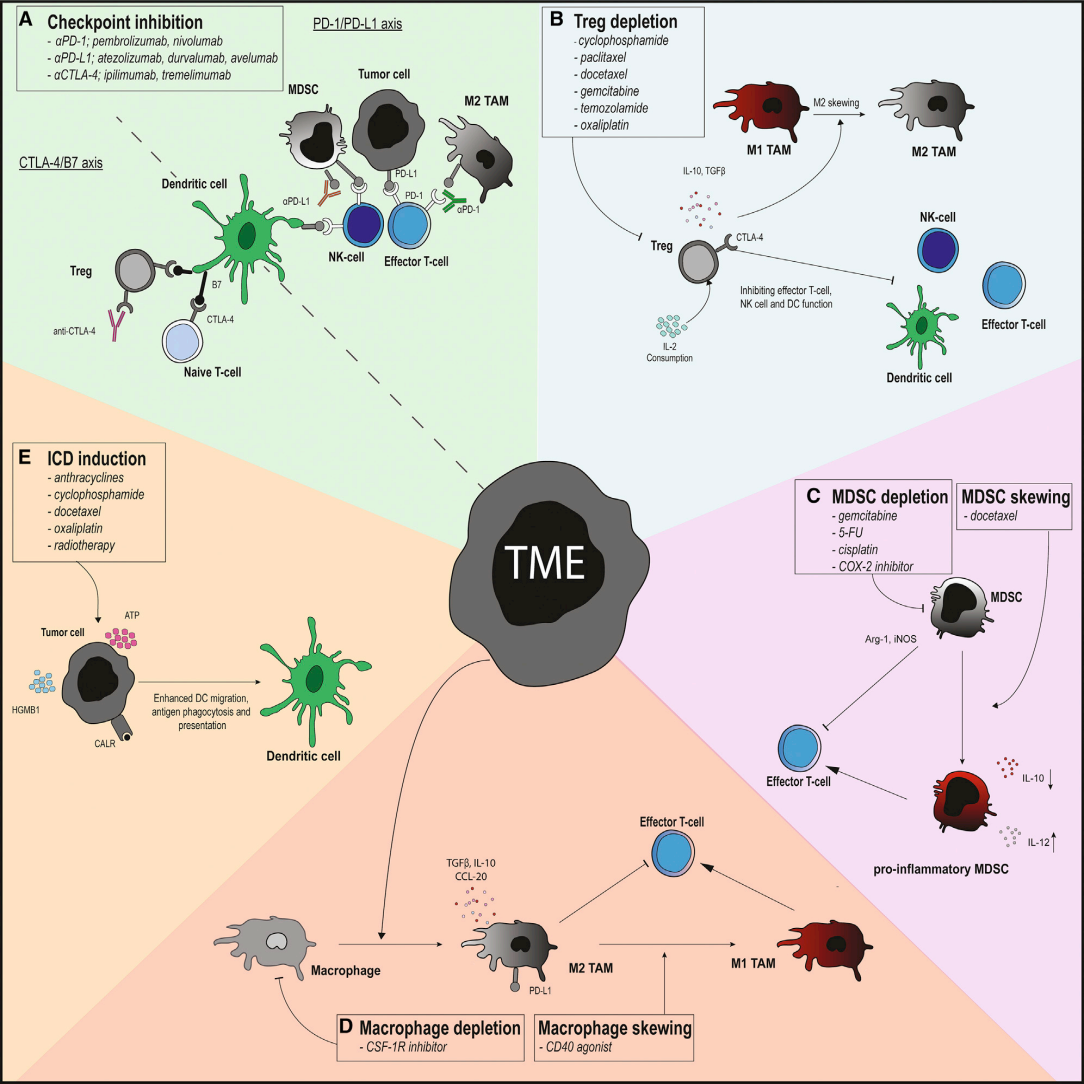
Targeting the TME with Conventional Treatment Modalities (A) Inhibitory molecules (PD-(L)1, CTLA-4) inhibit T-cell effector, dendritic cell and natural killer (NK)-cell function, and T-cell activation in the lymphnode. Checkpoint inhibitors targeting (PD-(L)1, CTLA-4) can reinvigorate the anti-tumor immune response induced by dendritic cell (DC) therapy by blocking PD-(L)1 signaling in the tumor and CTLA-4 in the lymph node. (B) Regulatory T cells (Tregs) exert their immunosuppressive mechanisms through inhibitory molecules (CTLA-4), secretion of immunosuppressive cytokines (interleukin [IL]-10, TGFβ), and IL-2 consumption, thereby inhibiting NK-cells, T cells, and DCs and skewing tumor-associated macrophages (TAMs) in a unfavorable M2 phenotype. Tregs can be depleted with several chemotherapeutics (cyclophosphamide, paclitaxel, docetaxel, gemcitabine, temozolamide, and oxaliplatin). (C) Myeloid-derived suppressor cells (MDSCs) can exert their immunosuppressive function by relieving Arginase 1 (Arg1) and inducible nitric oxide synthase (iNOS) to deprive T cells of metabolites. MDSCs can be depleted by chemotherapeutics gemcitabine, 5-FU, cisplatin, and docetaxel and skewed into a M1 phenotype by docetaxel. (D) M2 TAMs secrete IL-10 and transforming growth factor β (TGF-β) and are involved in tissue remodeling, wound healing, and tumor progression. M2 TAMs can be depleted by CSF-1R and skewed into an M1 phenotype by CD40 agonists. (E) Immunogenic cell death (ICD) is characterized by secretion of ATP and high mobility group box 1 (HGMB-1) and expression of Calreticulin (CRT) on the cell surface, which stimulates DC phagocytosis, antigen presentation, and migration. ICD can be induced by chemotherapeutics, cyclophosphamide, oxaliplatin, paclitaxel, docetaxel and anthracyclines, and radiotherapy.

Therefore, targeting these immunosuppressive features of the TME using FDA-approved treatment modalities, such as chemotherapy, radiotherapy, or more recently developed checkpoint inhibitors (CIs), in combination with DC therapy could improve DC therapy efficacy^1^, ^7^, ^8^, ^12^, ^14^, ^15^, ^16^, ^17^ (Figure 1). In this review, we discuss the immunological barriers that DC therapy faces and potential synergistic immunomodulating treatment modalities. In addition, we review clinical trials that have combined DC therapy with additional treatments. Data regarding these conducted clinical trials were found using a search string of relevant terms, as described in the Supplemental Information.

### Immunosuppressive Mechanisms of the TME and Tumor Cells that Hamper the Efficacy of DC Therapy

Both tumor cells and immunosuppressive immune cells in the TME hamper the effectivity of DC therapy through various mechanisms, such as the expression of inhibitory molecules, secretion of inhibitory cytokines or enzymes, induction of tolerogenic cell death, and creation of a dense extracellular matrix.^18^, ^19^ Tumor cells recruit immunosuppressive immune cells, fibroblasts,^20^ and endothelial cells to the TME through the secretion of growth factors, chemokines, and cytokines, thereby hampering the infiltration of DCs and other pro-inflammatory cells into the TME.^21^, ^22^ Moreover, fibroblasts and immunosuppresive immune cells interact synergistically with each other to maximize the immunosuppressive character of the TME.

#### Tolerogenic and Immunogenic Cell Death

Cancer cell death can be tolerogenic or immunogenic depending on the stimulus of apoptosis.^23^ Immunogenic cancer cell death leads to the secretion of DAMPs, attracts pro-inflammatory cells, and subsequently elicits a tumor-specific immune response (Box S1). Non-immunogenic cell death of malignant cells occurs without secretion of pro-inflammatory DAMPs. Tumor cells undergo non-immunogenic cell death through chemo-attraction of immunosuppressive phagocytes and induction of immunosuppressive phagocytosis.^24^ Tumor cells actively impair DC maturation through the secretion of immunosuppressive cytokines, leading to the presentation of tumor-associated antigens (TAAs) by immature DCs. Presentation of antigens by immature DCs induces T cell anergy and activation of TAA-specific regulatory T cells (Tregs), resulting in TAA-specific tolerance.^1^, ^25^, ^26^, ^27^

#### Tregs

Tregs are recruited to the TME through CCR4 chemokine signaling, and they expand in the TME upon transforming growth factor β (TGF-β) and interleukin (IL)-10 exposure.^28^ Tregs enable tumor progression by suppressing tumor-specific immune responses. In general, Tregs induce immunosuppression directly through cell-cell contact via inhibitory receptors, such as programmed cell death 1 (PD-1) or cytotoxic T-lymphocyte associated protein 4 (CTLA-4), or indirectly through the secretion of immunosuppressive cytokines, such as IL-10 and TGF-β, or IL-2 consumption of pore-forming proteins, such as granzyme and perforin.^29^ Via these mechanisms, Tregs suppress a wide array of pro-inflammatory immune cells that can be induced upon DC therapy, such as CD8^+^ T cells, CD4^+^ T cells, natural killer (NK) cells, NK T cells, and B cells. Additionally, Tregs can also suppress macrophages and DCs, thereby hampering the induction of an initial anti-tumor immune response.^8^, ^14^ Moreover, tumor-infiltrating Tregs have a higher affinity to TAAs derived from self-antigens presented on tumor cells than CD8^+^ T cells, thereby affecting the activation of TAA-specific CD8^+^ T cells in the TME.^22^ The Treg functions create a hostile and competitive environment for DC therapy-induced tumor-specific CD8^+^ T cells, hindering their cytotoxic functions.

#### Myeloid-Derived Suppressor Cells

Myeloid-derived suppressor cells (MDSCs) are immature myeloid cells with immunosuppressive effects. MDSCs are one of the most abundant immune cells in the TME, and they are attracted to the tumor site by chemokines secreted by the tumor cells.^30^, ^31^, ^32^ MDSCs comprise two subsets: monocytic MDSC (mMDSC) and polymorphonuclear MDSC (pMDSC).^33^, ^34^ mMDSCs tend to be more immunosuppressive than pMDSCs, as they are capable of both antigen-dependent and antigen-independent inhibition of T cell responses.^18^, ^35^ The hypoxic environment of the TME induces the release of hypoxia-inducible factor 1-alpha (HIF-1α), which causes mMDSCs to upregulate the enzymes arginase 1 (Arg1) and inducible nitric oxide synthase (iNOS) that break down L-arginase.^35^ Two products of this enzymatic reaction, urea and nitric oxide (NO), induce T cell depletion and inhibit T cell function.^18^, ^31^, ^36^, ^37^ Moreover, mMDSCs attract Tregs through C-C motif chemokine ligand 2 (CCL4) and CCL5 production, secrete IL-10,^38^ and upregulate PD-L1 on their cell surface, which inhibits tumor-specific T cell cytotoxicity.^39^ Furthermore, HIF-1α induces differentiation of mMDSCs into tumor-associated macrophages (TAMs) through the downregulation of phosphorylated signal transducer and activator of transcription 3 (pSTAT3), indicating that the TME can influence both immune cell function and differentiation.^40^, ^41^

#### TAMs

Monocytes are derived from the bone marrow and are recruited to the TME through CCL2 signaling, where these cells can differentiate into macrophages. Phenotypically, macrophages can broadly be divided into two subtypes: a pro-inflammatory M1 phenotype and an immunosuppressive M2 phenotype. Differentiation of M1 macrophages is induced by pro-inflammatory cytokines such as interferon (IFN)-γ and bacterial components such as lipopolysaccharide (LPS). M1 macrophages secrete pro-inflammatory cytokines, interleukins such as IL-12, and tumor necrosis factor α (TNF-α), leading to inflammation.^19^ Macrophages are skewed into an M2 phenotype, through the secretion of immunosuppressive cytokines by tumor cells or immune cells in the TME, and they inhibit CD8^+^ T cell function.^41^ TAMs display an M2-like phenotype and secrete IL-10, prostaglandin E2 (PGE2), and chemokines to attract and induce Tregs.^18^, ^41^ Moreover, TAMs express iNOS and Arg1, and they upregulate PD-L1 on their cell surface, which inhibits CD8^+^ T cell function.^18^, ^42^ These mechanisms hamper DC therapy-induced anti-tumor immunity.

### DC Therapy

#### Different DC Therapy Strategies

DC therapy aims at eliciting a tumor-specific immune response, by *in vitro* loading DCs *in vitro* with tumor antigens and additional maturation stimuli. DC therapy can be historically divided into three categories: first-, second-, and next-generation DC therapy.^1^ In first- and second-generation DC therapy, monocyte-derived DCs (moDCs) were used. moDCs are generated from monocytes upon culture with granulocyte-macrophage colony-stimulating factor (GM-CSF) and IL-4. moDCs have been shown to promote T cell differentiation and CD8^+^ T cell activation.^43^ In first-generation DC therapy, moDCs were loaded with a tumor lysate, TAAs, or synthetic peptides without additional maturation stimuli. Not surprisingly, without a proper maturation stimulus, clinical results were disappointing, with a tumor regression rate of 3.3%.^44^

In second-generation DC therapy, moDCs were additionally matured after loading these immature moDCs, using maturation cocktails, including IL-6, TNF, IL-1β, PGE2, and polyinosinic:polycytidylic acid (poly(I:C)).^45^ Maturing these tumor antigen-loaded moDCs significantly improved clinical results, with overall response rates (ORRs) of 8%–15%, depending on the tumor type.^9^ Median overall survival (OS) was increased by 20% in multiple clinical trials with second-generation DC therapy, which is the threshold for clinical relevance.^9^, ^45^, ^46^ Furthermore, the IMPACT trial showed an increase in median OS of 3.9 months for castration-resistant prostate cancer patients treated with sipuleucel-T (DC therapy) compared to the placebo group, leading to FDA approval in 2010.^47^

In next-generation DC therapy, naturally occurring DCs (nDCs), such as plasmacytoid DCs (pDCs) and conventional DCs (cDCs), are used for vaccination. cDCs can be divided into two main subtypes: cDC1 and cDC2.^48^ cDC1s are superior in cross-presenting antigens and, thereby, inducing CD8^+^ T cell activation. Recent studies have shown that cDC1s are critically important for anti-tumor immune responses and that their presence in the TME positively correlates to OS and clinical responses upon PD-1 monoclonal antibody (mAb) in melanoma.^49^, ^50^, ^51^ Classically, cDC2s mainly activate CD4^+^ T cells. The characterization of human cDC2 function remains difficult, as this subset is very heterogeneous, shares markers such as CD11b and CD172a with macrophages and moDCs, and has functional overlap with cDC1s.^50^ pDCs in the TME produce type 1 IFN, which attracts NK, B, and T cells.^52^ However, pDCs have questionable antigen-presenting skills, as CD123^+^ pDCs were found to be contaminated with pre-cDCs.^53^ Naturally occurring DCs can, therefore, be selected based on their superior functional properties, and they can be obtained without an additional culture period, leading to reduced production costs.^1^

DCs can also be targeted *in vivo* using Toll-like receptor (TLR) ligands, intra-tumoral injection of TriMix mRNA, or attenuated viral agents (virotherapy).^54^, ^55^, ^56^ Furthermore, FMS-like tyrosine kinase 3 ligand (FLT3L) injection increases cDC proliferation and infiltration into the tumor site, which enhanced PD-L1 mAb efficacy in a melanoma mouse model.^57^, ^58^ Clinical trials exploiting next-generation DC therapy are currently being performed, and the coming years will indicate whether the use of nDCs further improves the clinical efficacy of DC therapy.^59^, ^60^ Currently, clinical studies comparing the effectivity of different DC subtypes for vaccination purposes are lacking and are urgently needed to determine which DC subset would induce the most effective anti-tumor immune response.^61^

#### Immune Monitoring

Immunological responses induced by DC therapy are generally measured by IFN-γ enzyme-linked immunospots (ELISPOTs), tetramer analysis, co-cultures with lysate-loaded DCs, and delayed type hypersensitivity (DTH) skin tests. Correlating these immunological parameters to clinical response remains challenging, as not all patients show increased IFN-γ production in an ELISPOT upon DC therapy, and even positive ELISPOTs can be encountered before DC therapy.^62^, ^63^ Furthermore, tumor-specific IFN-γ production by peripheral blood mononuclear cells (PBMCs), as determined by an ELISPOT analysis, often does not correlate with clinical outcome or allergic reaction measured by the DTH skin test.^62^, ^64^, ^65^ However, in some studies, immunological parameters have been correlated with clinical parameters in both hematological and solid malignancies.^59^, ^66^, ^67^

DTH skin tests have been shown to correlate with clinical outcome in DC therapy trials in melanoma and colorectal cancer patients.^68^, ^69^, ^70^ Furthermore, in mesothelioma patients treated with DC therapy, two patients with a negative DTH skin test had progressive disease and had the shortest OS,^71^ indicating that the DTH skin test could correlate with clinical outcomes. Additionally, in pancreatic cancer patients treated with Wilms tumor protein 1 (WT1) I-II peptide-loaded DC therapy, positive DTH skin tests correlated with longer progression-free survival (PFS) and OS than negative DTH skin tests.^72^ However, all other studies combining DC therapy and chemotherapy did not show any correlation between DTH skin testing and clinical outcome.^62^, ^64^, ^65^, ^72^, ^73^, ^74^ The lack of correlation between DTH skin tests with clinical outcome could be dependent on the timing of the DTH skin test, the evaluation of response to the skin test, and the lack of a negative control. The classification of a positive DTH skin test varies from 2- to 5-mm erythema, while one could argue that induration is a more important parameter.^75^ Also timing of DTH skin testing varies between studies or is not mentioned.

### Combination Strategies to Optimize DC Therapy

#### Combining CIs with DC Therapy

Inhibitory molecules that hamper anti-tumor immune responses, such as PD-1, PD-L1, and CTLA-4, can be expressed on both tumor cells and various immune cells.^76^, ^77^ Blocking these inhibitory molecules has been shown to restore tumor-specific T cell activity.^76^ There are many other inhibitory and even co-stimulatory molecules identified that could function as potential targets for immunotherapy, such as lymphocyte activation gene-3 (LAG-3), B and T lymphocyte attenuator (BTLA), programmed death-1 homolog (PD-1H), T cell immunoglobulin (TIM-3), T cell immunoglobulin and immunoreceptor tyrosine-based inhibitory motif domain (TIGIT), glucocorticoid-induced TNF receptor (GITR), and NK cell inhibitory receptor NKG2A.^78^, ^79^, ^80^, ^81^, ^82^ The efficacy of these co-inhibitory and co-stimulatory molecules is currently being investigated in preclinical and/or clinical studies, and it is, therefore, not addressed in this review.

CTLA-4 blockage inhibits T cell activation in the lymph node, whereas blocking the PD-1 and PD-L1 axis mainly inhibits the effector function of activated T cells in the TME.^83^ Anti-CTLA-4 (ipilimumab and durvalumab), anti-PD-L1 (atezolizumab, durvalumab, and avelumab), and anti-PD-1 (nivolumab and pembrolizumab) are registered for the treatment of solid tumors, because of striking clinical effects. The efficacy of these CIs, especially PD-(L)1 mAb, often depends on and correlates with PD-L1 expression in the TME, mutational burden, and the number of tumor-infiltrating lymphocytes (TILs).^13^, ^84^, ^85^, ^86^, ^87^ High ORRs of 57% are reported in immunogenic cancers such as melanoma, which is ascribed to a high mutational burden and high numbers of TILs.^88^, ^89^ In tumors with lower mutational burden (e.g., mesothelioma), ORRs remain between 9% and 25%,^90^ likely due to the relative low frequency of TILs. DC therapy induces the infiltration of tumor-specific CD8^+^ T cells and upregulates PD-1 expression on these TILs, which could render tumors with low TIL numbers susceptible to anti-PD-(L)1 treatment.^91^, ^92^, ^93^, ^94^

It is likely that the limited efficacy of DC therapy trials is in part due to inhibitory signaling in the TME and lymph node. Additional administration of CIs can block inhibitory signaling on tumor cells and immunosuppressive cells in the TME. CIs could even inhibit the signaling of these inhibitory molecules on the DCs administered during DC therapy, as DCs express PD-1, PD-L1, and PD-L2.^95^ Expression of PD-1 and its ligands likely limits the induction of tumor-specific immune responses, as high PD-L1 expression on DCs suppresses CD4^+^ and CD8^+^ T cell proliferation and promotes Treg proliferation in various diseases, including cancer.^8^, ^96^, ^97^, ^98^, ^99^, ^100^, ^101^ This suggests that combining DC therapy with CIs can result in a two-sided synergy by targeting not only tumor cells and immunosuppressive cells in the TME but also DCs administered during DC therapy and even T cells induced by DC therapy (Figure 1A). Different DC subtypes differentially express these inhibitory molecules, suggesting that specific CIs should be used in combination with certain DC subsets.^102^ The rationale for using CIs in combination with DC therapy is further supported by the finding that the addition of a PD-1 mAb to *ex vivo*-cultured autologous T cells and DCs of patients with myeloma improved IFN-γ production while limiting Treg expansion.^103^ Furthermore, combining DC therapy with systemic PD-1 blockade in mice bearing intracranial glioma tumors improved survival compared to both single treatments.^91^ In addition, PD-1 blockage on DCs, administered in a breast tumor-bearing mouse model, that were subsequently systemically treated with anti-PD-1 mAb reduced tumor growth and increased survival compared to untreated mice.^104^

#### Clinical Trials Combining CIs with DC Therapy

Until now, three clinical studies combined DC therapy with CI treatment. In all of these studies, CTLA-4 mAb has been used. Clinical responses were retrospectively observed in patients with stage III and IV melanoma that were treated with ipilimumab upon disease progression, after receiving at least 3 bi-weekly vaccinations with gp100 and tyrosinase-loaded DCs.^105^ Especially patients with stage III melanoma responded well, with an OS rate of 51% after 2 years. The presence of tumor-specific T cells obtained from DTH skin biopsies did not correlate to OS in patients with stage III and IV melanoma. Ipilimumab-related adverse events were not increased in patients pretreated with DC vaccination (58%) compared to patients treated with ipilimumab monotherapy (61%–70%).^106^

In a clinical phase I, dose escalation trial, patients with stage IIIc or IV melanoma received three bi-weekly intradermal vaccinations with MART-1-loaded DCs and concurrent systemic treatment with a dose escalation of tremelimumab (3, 6, and 10 mg/kg), a CTLA-4-blocking mAb.^107^ Four of 16 patients developed a clinical response upon treatment, of which 2 patients developed a complete response (CR) and 2 patients developed a partial response (PR). This indicates that response rates upon combination therapy are promising, compared to response rates to tremelimumab monotherapy (7%–10% ORR)^107^, ^108^, ^109^, ^110^ and DC vaccination monotherapy (15% ORR).^9^ Remarkably, responses were also observed in patients treated with only 3 mg/kg tremelimumab, achieving plasma levels of 30 μg/mL, which is below the target level determined by prospective clinical trials.^108^ This suggests that these results are not solely the effect of tremelimumab or DC therapy alone, indicating a synergistic effect. However, immunological analysis was inconclusive, with a minority of patients showing a response to tetramer or ELISPOT analysis.^108^

A phase II, open-label, single-arm clinical trial combined ipilimumab treatment with DCs electroporated with Trimix-mRNA (CD40L, CD70, constitutive active TLR4) and mRNA encoding for MAGE-A3, MAGE-C2, tyrosinase3, or gp100 in patients with stage III and IV melanoma.^111^ Radiological responses were assessed with immune-related response criteria (irRCs), which showed an ORR of 38%; 20% of the responding patients showed a CR and 18% had a PR. A disease control rate of 51% at 6-month follow-up was observed, and the ORR was better than ORRs observed in patients treated with ipilimumab as monotherapy.^112^ Furthermore, the number of CRs was similar to clinical trials investigating combination therapy of ipilimumab and nivolumab.^106^ Immunological analysis showed an overall increase of CD4^+^ and CD8^+^ T cells in the peripheral blood and a positive tumor antigen-specific ELISPOT analysis in two of ten patients.^111^

Combining different CIs often leads to increased toxicity, such as dermatologic toxicity, colitis, or pneumonitis,^113^, ^114^ whereas combining DC therapy with CIs does not increase the immune-related adverse event profile of CI monotherapy. Furthermore, ORRs, PFS, and OS in clinical trials investigating combination therapy consisting of DC therapy and CTLA-4 mAb treatment are promising, as compared to clinical trials that investigated these therapies as monotherapy. However, phase III, randomized, controlled clinical trials are needed to determine the efficacy improvement of combining DC therapy and CI to either treatment modality alone. Furthermore, combining PD-(L)1-targeting CIs with DC therapy still needs to be evaluated in clinical trials. Immunological analysis was inconclusive in all studies and did not correlate with clinical outcome. Concurrent intensive immunological analysis of blood and tumor material could provide proof of principle, expand current knowledge, and possibly lead to objectifiable immunological parameters for immunotherapy. Currently, many phase I-II trials are being conducted that combine PD-(L)1 mAb with DC therapy.^56^, ^115^ However, to date, there are no clinical phase III trials being conducted to observe the synergy of CI treatment in combination with DC therapy (https://clinicaltrials.gov/).

#### Combining Chemotherapy with DC Therapy

Apart from specifically targeting cancerous cells, it is becoming apparent that chemotherapy can also actively influence the immune system by depletion of specific cell types, such as Tregs and MDSCs, and by induction of immunogenic cell death (ICD). Furthermore, chemotherapy can skew immunomodulatory cells in a more pro-inflammatory subset. Depletion of Tregs^12^ and MDSCs^116^ in the TME after chemotherapy treatment was already observed in preclinical and clinical studies.^11^, ^12^, ^17^, ^23^, ^117^, ^118^ Such immunological changes were even associated with clinical response.^118^, ^119^, ^120^, ^121^, ^122^ Furthermore, ICD-inducing chemotherapy, such as anthracyclines, has a suboptimal result in immunodeficient mice.^23^, ^123^ This indicates that chemotherapy treatment can affect the immunosuppressive TME by cell-specific depletion and improve immune responses by the induction of ICD, thereby proving beneficial when combined with DC therapy.

In most clinical trials, DC therapy was administered in combination with registered chemotherapy treatment. Therefore, studies that combined treatment of chemotherapeutics with DC therapy were not often designed to improve DC therapy efficacy. Consequently, most of the immunological parameters, such as IFN-γ ELISPOTs, tetramer analysis, and DTH skin tests, determined immunological response to DC therapy rather than the immunological effects induced by chemotherapy.^62^, ^63^, ^65^, ^72^, ^124^, ^125^ Consequently, monitoring of immunomodulatory effects and, therefore, objectifying the attributable effect of chemotherapy on DC therapy is difficult to achieve. Clinical trials that investigated treatment with DC therapy in combination with chemotherapy in esophageal, prostate, and pancreatic cancers; mesothelioma; glioblastoma; and melanoma are summarized in Table 1.

**Table 1:** Overview of Clinical Trials Combining moDC Therapy with Chemotherapy

Disease	Loading Material for DCs	Maturation Cocktail	CTX (+ Other Additional Treatments)	Immunomodulatory Effect^a^	Immunological Rationale^b^	CTX Immune Readout^c^	DC Therapy Immune Readout d	n	CO	CO Corresponding to IR	Reference
**Glioblastoma**	autologous tumor lysate	TNF-a, IFN-a and POLI I:C	RTX + TMZ 75 mg/m2TMZ 200 mg/m2	Treg depletion	none	none	8/25 pos ELISPOT	31	PFS 12.7m, OS 23.4m	no	^63^
**Pancreatic cancer**	WT1 peptide	OK-432 and PGE2	S1 or S1 + gemcitabine dose not stated	Treg depletion MDSC depletion	none	none	7/8 pos ELISPOT	8	4 PD 4 alive 2 years post-treatment	pos ELISPOT correlated to 2-year OS	^125^
**Pancreatic cancer**	WT1-I, -II, I/II peptide	OK-432 and PGE2	gemcitabine 1000 mg/m2	Treg depletion, MDSC depletion	none	none	4/11 pos DTH	10	7 SD, 3 PD	pos DTH positively correlated with PFS	^72^
**Glioblastoma**	autologous tumor lysate	monocyte-derived conditioned medium (MCM)	TMZ 150-200 mg	Treg depletion	none	none	2/9 pos ELISPOT 0 pos DTH	14	2 PR, 3 SD, 4 PD median OS 23m	no	^62^
**Esophageal cancer**	WT1 peptide	OK-432 andPG-E2	DTX 50 mg/m2	Treg depletion, MDSC skewing, ICD induction	non-specific immune enhancement	none	5/8 pos ELISPOT 3/7 pos DTH 3/7 pos tetramer 5/8 pos HLA	10	10 PD	no	^65^
**Melanoma**	WT1 peptide, gp100 tyrosinase, MAGE-A3 or MAGE-A2	Matured with OK-432 and PG-E2	carboplatin (AUC 5) and paclitaxel (175 mg/m2)	Treg depletion	Treg depletion and decrease IL-10 and TGF-β secretion	none	4/9 pos ELISPOT	9	1 PR, 4 SD, 5 PD OS 12m PFS 2,3m	no	^124^
**Prostate cancer**	PSA, PAP mRNA	TNF-a IL-1B, IL-6, PGE2	DTX 75mg/m2	Treg depletion, MDSC skewing, ICD induction	MDSC depletion and ICD	DC + DTX: decrease in MDSC	9/18 pos ELISPOT 5/18 pos DTH	19 DTX 21 DTX + DC	no difference in PFS and OS	decreasing levels of MDSC were correlated to better PFS	^64^
**Melanoma**	p53, survivin, and hTERT mRNA	TNF-a IL-1B, IL-6, PGE2	cyclophosphamide 50 mg	Treg depletion, ICD induction	Treg depletion	CD4^+^ T-cell depletion	6/17 pos ELISPOT	22	9 SD, 13 PD OS 10,4m PFS 3,1m	no	^127^
**Mesothelioma**	autologous tumor lysate	PGE2 TNF-a IL-1B IL-6	cyclophosphamide 2x50 mg	Treg depletion, inducing ICD	Treg depletion	Treg depletion	8/10 pos DTH	10	1 CR, 4 SD, NA 3,PD 2	no	^71^
**Prostate cancer**	killed LNCaP prostate cancer cells	poly I:C	50 mg cyclophosphamide, 75 mg/m2 DTX	Treg depletion, MDSC skewing, ICD induction	Treg depletion, enhancement of T and NK cell activation	Treg depletion	increased CD8^+^ T-cells, increased PSA- specific IFN- γ production	24	OS 19m	no	^132^
**Melanoma**	autologous pulsed DC	TNF-a IL-1B, IL-6 PGE2	TMZ 75mg/m2 (IL-2)3,000,000 IU/day	Treg depletion	Treg depletion	Treg depletion	9/17 pos DTH	17	1 PR, 6 SD, 10 PD	no	^74^
**Melanoma**	autologous tumor lysate or survivan, hTERT, p53	TNF-a IL-1b IL-6 PGE2	IL-2, cyclophosphamide and a COX-2 inhibitor	Treg depletion, MDSC depletion	Treg depletion, MDSC depletion	Treg increase, MDSC depletion	8/17 pos DTH at baseline 1/17 pos DTH after vaccination	28	16 SD, 12 PD PFS 4,5m	no	^73^

DC, dendritic cell; CTX, chemotherapy; CO, clinical outcome; IR, immunological readout; n, number; LNCaP, androgen-sensitive human prostate adenocarcinoma cell line; WT, Wilms tumor; MAGE, melanoma-associated antigen; PSA, prostate specific antigen; PAP, prostate acidic phosphatase; p53, tumor protein p53; hTERT, telomerase reverse transcriptase; gp100, glycoprotein 100; RTX, radiotherapy; S-1, Tegafur/gimeracil/oteracil; TMZ, temozolomide; DTX, docetaxel; COX, cyclooxygenase; IL, interleukin; Treg: regulatory T cell; MDSC, myeloid derived suppressor cell; ICD, immunogenic cell death; PFS, progression free survival; m, months; OS, overall survival; PR, partial response; SD, stable disease; PD, progressive disease; ELISPOT, enzyme-linked immunospot assay; DTH, delayed type hypersensitivity skin test; pos, positive; AUC, area under the curve; NA, not applicable because of non-measurable lesions; TGF- β, transforming growth factor β; OK-432, penicillin-killed and lyophilized preparations of a low virulence strain (Su) of Streptococcus pyogenes.

a The hypothesized immunomodulatory effect of chemotherapeutics as described in preclinical studies and reviews.

b The immunological rationale for the use of the chemotherapeutic agent described in the respective article.

c The results of the immunological analysis done to evaluate immunomodulatory effects of chemotherapeutics.

d The results of the immunological analysis done to evaluate immunomodulatory effects of DC therapy.

#### Targeting of the Immunosuppressive Environment by Chemotherapy

##### Tregs

Various chemotherapeutics, such as cyclophosphamide, paclitaxel, docetaxel, gemcitabine, temozolamide (TMZ), and oxaliplatin, are capable of Treg depletion in clinical and preclinical settings.^12^, ^17^, ^117^, ^118^, ^126^ Cyclophosphamide is the best known and studied chemotherapeutic agent with the capability of depleting Tregs. Four clinical studies evaluated the immunological and clinical effects of potentially Treg-depleting chemotherapeutics in combination with DC therapy, in which 3 studies used cyclophosphamide^64^, ^71^, ^73^ and one study used TMZ.^74^ Two other studies evaluated only clinical effects of potentially Treg-depleting chemoteherapeutics, cyclophosphamide and paclitaxel, in combination with DC therapy.

In a phase I clinical trial, melanoma patients were treated with six biweekly injections of DCs electroporated with mRNA encoding p53, survivin, and hTER and concurrent low-dose cyclophosphamide (2 × 50 mg/day biweekly).^127^ The OS was 10.4 months and 9 of 22 patients had stable disease (SD). Tregs as well as total CD4^+^ T cells were depleted upon cyclophosphamide treatment, questioning whether cyclophosphamide induced specific depletion of Tregs.^127^ However, another clinical trial in mesothelioma patients that also combined concurrent low-dose cyclophosphamide (2 × 50 mg/day biweekly) with three biweekly injections of DCs loaded with tumor lysate did show selective depletion of Tregs.^71^ Unfortunately, depletion of Tregs was not correlated with a better clinical outcome. However, detailed analysis of naive Tregs (nTregs, CD45RA^+^ FoxP3^int^) and activated Tregs (aTregs, CD45RA^−^ FoxP3^hi^) showed a positive correlation between the pretreatment levels of nTregs and OS.^128^ In addition, results from this clinical study are quite promising, with patients still alive up to 6 years after diagnosis.

Another phase II clinical study combined DC therapy loaded with tumor lysate or peptides (surviving, telomerase and p53) with consecutive IL-2 (2 million international units [mIUs]/day for 5 days) with metronomic cyclophosphamide (2 × 50 mg/day biweekly) and a Cox-2 inhibitor (200 mg daily) in melanoma patients.^73^ Melanoma patients treated with this combination therapy also showed increased numbers of Tregs after four vaccinations, indicating that cyclophosphamide was not able to counteract the effect of IL-2 on Tregs, as IL-2 has the potency to increase Treg numbers.^129^ In contrast to Tregs, mMDSCs were significantly decreased after four vaccinations, indicating that the combination treatment depletes mMDSCs. However, the changes observed in Treg numbers and mMDSC frequency did not correlate with clinical outcome. Clinical results were significantly improved compared to a previous trial where DC therapy was only combined with IL-2.^130^

Combining neoadjuvant TMZ (75 mg/m^2^/day for 14 days) treatment followed by autologous tumor lysate-loaded DC therapy with consecutive IL-2 (3 mIU/day for 5 days) in 17 melanoma patients, also significantly depleted Tregs, although this did not correlate to clinical outcome. One patient showed a PR and six patients had SD.^74^

In another study, patients with metastatic castration-resistant prostate cancer were treated with neoadjuvant metronomic cyclophosphamide (50 mg/day for 1 week) followed by LNCaP- (androgen-sensitive human prostate adenocarcinoma cells) loaded DC therapy and docetaxel (75 mg/m^2^ every 3 weeks). The predicted OS was 11.3 months, whereas in this study an OS of 19 months was observed, suggestive of a synergistic effect upon the combination of these treatments.^131^, ^132^

In a clinical trial in patients with stage IV melanoma treated with DCs loaded with a multi-peptide (WT1, gp100, tyrosinase, and MAG-E3 or MAGE-A2) combined with paclitaxel (175 mg/m) and carboplatin (area under the curve 5), an OS of up to 24 months was observed.^124^ Unfortunately, Treg numbers were not assessed in these clinical trials. Taken together, these clinical trials indicate that chemotherapy is capable of depleting Tregs and inducing promising clinical responses in combination with DC therapy. Alterations in Treg numbers upon treatment could not be correlated with clinical outcome, although nTreg frequencies at baseline were predictive of clinical response.^71^, ^73^ However, further research is needed to determine whether this is also observed in other malignancies and combination therapies (Figure 1B).

##### MDSCs

Numerous chemotherapeutics, such as gemcitabine, 5-FU, cisplatin, and docetaxel, have been shown to specifically deplete MDSCs.^12^, ^73^, ^117^, ^118^ Furthermore, docetaxel possibly improves immunostimulatory effects of total MDSCs by skewing them into a more favorable pro-inflammatory and migratory phenotype (CCR2, CCR5, CX3CR1, and CCR7) rather than an immunosuppressive phenotype.^133^ This indicates that chemotherapeutics not only deplete immunoregulatory cells but also can change the phenotype of immunosuppressive cells. In contrast to the above-described chemotherapeutics, cyclophosphamide increases the amount of specific pMDSCs, but not mMDSCs, in the peripheral blood of mice and human.^134^ The increase in pMDSCs induced by cyclophosphamide can be counteracted by the addition of chemotherapeutics targeting MDSCs, as combining cyclophosphamide and gemcitabine treatment decreased both Treg and GR1^high^ MDSC numbers and reduced tumor growth.^135^

One study in patients with stage IV pancreatic ductal adenocarcinoma treated with WT1-loaded DC therapy and gemcitabine (1,000 mg/m^2^ three times every 28 days) showed that combining these treatments is safe and feasible.^72^ They also found a positive correlation between DTH skin testing and PFS. Unfortunately, MDSC numbers were not assessed in this study, so it remains inconclusive whether gemcitabine administration in combination with DC therapy affected MDSC numbers in these patients.

In a clinical study in patients with metastasized adenocarcinoma of the prostate, docetaxel (75 mg/m^2^ every 3 weeks) monotherapy was compared to combined treatment of docetaxel with DCs transfected with mRNAs encoding PAP (prostate acidic phosphatase) and PSA (prostate-specific antigen).^64^ There was no significant difference in OS and PFS between both treatment arms. Patients with decreased MDSC frequencies in cryopreserved PBMCs upon treatment had a longer PFS as compared to patients with increasing frequencies of MDSCs upon treatment. A decrease of MDSCs in cryopreserved PBMCs was only observed 6 weeks after the start of combination therapy, but not upon docetaxel monotherapy, which suggests that docetaxel monotherapy is not sufficient to decrease MDSCs in peripheral blood.^64^ Paradoxically, a preclinical study observed a decrease in total MDSC numbers upon treatment with docetaxel monotherapy.^133^ Additionally, docetaxel treatment skewed total MDSCs toward a more pro-inflammatory phenotype in a preclinical study (Figure 1C).^133^ Characterization of MDSCs remains challenging due to a lack of specific markers and the need to assess these cell populations in freshly isolated blood, as especially pMDSCs are lost upon cryopreservation.^33^ In addition, most clinical trials focus on evaluating MDSC numbers rather than MDSC characteristics, leading to a lack of evidence for the phenotypical switch of MDSCs upon docetaxel treatment in humans.

##### ICD

Various chemotherapeutics, such as cyclophosphamide, oxaliplatin, paclitaxel, docetaxel, and anthracyclines, are able to induce ICD (Figure 1E). The antineoplastic effects of these chemotherapeutics are also dependent on ICD.^136^, ^137^, ^138^, ^139^ Current monitoring of ICD occurs via vaccination assays that are not applicable in clinical trials.^139^ This limits the possibility for evaluation of ICD-induced synergistic effects. Consequently, ICD is not often used as a rationale for combining chemotherapy and DC therapy (Table 1). A phase III clinical study in patients with metastatic castration-resistant prostate cancer comparing docetaxel treatment combined with DC therapy and docetaxel monotherapy has finished; accrual end results are awaited (ClinicalTrials.gov: NCT02111577). Hopefully, immunological data will lead to a better understanding of the immunomodulatory effects of docetaxel. A phase III trial in glioma patients evaluates the additional effect of DC therapy to current treatment consisting of TMZ and radiotherapy (ClinicalTrials.gov: NCT03548571). The addition of a DC monotherapy arm to both these studies could have revealed the synergistic effect of docetaxel and TMZ on DC therapy.

#### Combining Radiotherapy with DC Therapy

Radiotherapy has been used as local tumor treatment for the last century. Recently, radiotherapy was also found to affect non-radiated tumor lesions, which is called the abscopal effect. This suggests systemic effects of radiotherapy that can be explained by the upregulation of radiation-induced double-stranded DNA in the cytosol, which serves as a DAMP for the instigation of ICD. Subsequently, the secretion of type I IFNs by tumor cells will attract cDC1s to the tumor site, which can engulf the released tumor antigens and initiate an immune response.^16^, ^140^ Therefore, radiation-induced ICD can act as *in situ* vaccination.

Apart from inducing ICD, radiation induces the upregulation of adhesion molecules on the vascular endothelium of tumor cells, which enables T cell infiltration^141^ (Figure 1E). Furthermore, a non-lethal radiotherapy dosage increases surface expression of first apoptosis signal (Fas) ligand, carcinoembryonic antigen, and major histocompatibility complex I (MHCI) on tumor cells, enabling tumor-specific CD8^+^ T cells to recognize the tumor cells and exert their cytotoxic effects.^142^ Together, these immunomodulatory effects are hypothesized to be responsible for the abscopal effect.^143^ However, the theoretical immunomodulatory effect of radiotherapy lacks clinical support, as a recent review found only 46 reported cases of abscopal effect from 1969 to 2004.^144^ This could be dependent on the irradiation dose used, radiation schedule, and lack of additional immunostimulation. This is supported by a recent study in breast and colorectal tumor-bearing mice, where different radiation doses were compared in combination with CI. Here they found that repetitive radiation at a low dose (5–8 Gy) was more effective than a high (20-Gy) single dose.^145^, ^146^ High-dose radiation is thought to indirectly downregulate cytosolic double-stranded DNA (dsDNA) and, thus, inhibit radiation-dependent ICD.^16^

#### Clinical Trials Combining DC Therapy with Radiotherapy

In a phase I clinical trial, 14 patients with advanced hepatoma received immature DC therapy followed by 8-Gy radiotherapy. The clinical results varied, with 2 PRs, 4 minor responses, 3 SD, and 4 PD.^147^ Seven of ten immunologically evaluated patients developed an IFN-γ ELISPOT response upon treatment, which did not correlate with clinical response.

In an observational study, patients with esophageal cancer receiving autologous tumor lysate-loaded DC therapy in combination with concurrent radiotherapy (60 Gy) were compared to radiotherapy alone. The 2-year survival was significantly improved upon combination therapy (67.8%) as compared to single radiotherapy treatment (33.3%).^148^ Additionally, in patients with metastatic solid tumors, radiotherapy (35 Gy) combined with *in situ* DC therapy using GM-CSF administration, induced an abscopal effect in 11 of 41 patients, which was significantly higher compared to abscopal effects induced by radiotherapy alone.^149^

These data suggest that regional radiotherapy can act synergistically when combined with DC therapy. The synergy likely depends on the release of tumor antigen that boosts the anti-tumor immune response, and it should be further investigated in ongoing clinical trials. A recently registered trial for patients with metastatic melanoma will evaluate the additional effect of different immunostimulatory agents, including radiotherapy (24–32 Gy), to autologous tumor lysate-loaded DC therapy (ClinicalTrials.gov: NCT01973322).^150^ Additionally, the effect of sipuleucel-T therapy combined with stereotactic ablative body radiation in patients with metastatic castration-resistant prostate cancer will be observed in a single-arm study (ClinicalTrials.gov: NCT01818986). These studies will allow further investigation into the exact immunological mechanism of action of radiotherapy combined with DC therapy.

#### Other Combination Strategies: Targeting TAMs

Immunoinhibitory TAMs are abundant in many solid tumors.^151^, ^152^ These TAMs can be either depleted via colony-stimulating factor 1 receptor (CSF-1R) blockade or skewed into an immunostimulatory M1 phenotype by CD40 agonistic mAb.^153^, ^154^, ^155^, ^156^, ^157^, ^158^, ^159^ CSF-1R blockade increased the efficacy of chemotherapy in pancreatic tumor-bearing mice.^160^ In glioblastoma tumor-bearing mice, TAM depletion by CSF-1R monotherapy induced tumor reduction and increased survival.^161^ In contrast, in a mesothelioma mouse model, CSF-1R kinase inhibitor PLX3397 (pexidartinib)-mediated TAM depletion as monotherapy did not improve survival.^153^ However, when pexidartinib treatment was combined with DC therapy, improved survival was observed when compared to both monotherapies, which was accompanied by increased numbers of proliferating T cells and effector T cells.^153^

These studies indicate that TAM depletion can improve the immunosuppressive character of the TME and, thereby, act synergistically when combined with DC therapy. In addition to TAM depletion, skewing TAMs toward an immunostimulatory M1 phenotype using CD40 mAb may even hold more clinical potential, as CD40 mAb-activated macrophages in pancreatic cancer infiltrate the tumor and facilitate tumor stroma depletion.^162^ Especially in tumors with dense stroma, targeting the stroma indirectly through macrophage skewing could facilitate and improve tumor-specific T cell infiltration upon DC therapy (Figure 1D).

### Future Perspectives

Currently, most clinical trials that combine DC therapy with other treatments, such as chemotherapy, radiotherapy, or CI, often lack immunological rationale. This is likely due to already existing registrations of these treatments based on other rationales, leading to the mandatory use of certain therapies in specific dosages and schedules. In an experimental setting, a lack of consistency in dosing and schedule of treatments complicates the comparison of both immunological responses and clinical efficacy between different studies. For example, a “*metronomic*” dosage of cyclophosphamide varies from 2 × 50 mg/day to 100 mg/day between different studies,^71^, ^127^ whereas Treg depletion is dependant on the dose and schedule of chemotherapy.^29^ Separate studies should be performed to analyze the immunomodulatory effects of chemotherapy, radiotherapy, targeted therapies, and immunotherapies alone. During these studies, adequate reports of the used methods and analyzation strategies are necessary to facilitate the standardization of type and timing of immunomonitoring assays.^163^ This will create the possibility to replicate and subsequently compare the immunomodulatory effects between studies, and, finally, it leads to a better understanding of the immunomodulatory effects of monotherapies.^115^ With this knowledge, specific therapies with the right dosing can be combined with DC therapy based on the TME characteristics (Figure 1).

Variations in DC therapy, in terms of antigen loading, maturation, use of different DC subsets, dosage per injection, and the interval or total amount of vaccinations, make it difficult to compare clinical studies with each other. Adequate immunomonitoring in DC therapy is mandatory to create the possibility to compare studies and evaluate the effects of different antigen-loading methods, maturation cocktails, and administration schedules or injection sites. Additionally, registration of DC therapies will enable the investigation of clinical efficacy of DC therapy in combination with other treatments as compared to DC monotherapy. This will inevitably lead to an increase in the number of randomized trials and rapid release and approval for registration of immunomodulatory therapies in combination with DC therapy. To date, sipuleucel-T is the only registered DC therapy.^47^ Phase III trials that could eventually lead to the registration of DC therapy are ongoing for melanoma (ClinicalTrials.gov: NCT02993315), glioblastoma (ClinicalTrials.gov: NCT03548571), mesothelioma (ClinicalTrials.gov: NCT03610360), and colorectal cancer (ClinicalTrials.gov: NCT02503150).

Hopefully, trials combining DC therapy with other therapies, based on a solid rationale and performed with adequate immunomonitoring and uniformity in administration schedules, will lead to the registration of already existing treatment modalities for new purposes. In this way, chemotherapeutics, radiotherapy, CIs, or other targeted therapies can be used as off-the-shelf, affordable immunomodulating agents to support DC therapy in a personalized manner. To accomplish this, intensive cooperation between clinicians and basic scientists will be needed.

Other targets for cancer therapy, such as additional co-inhibitory molecules, co-stimulatory molecules, or even targeted therapies such as indoleamine 2,3-dioxygenase (IDO), are upcoming. These targets could all hypothetically be used as immunomodulators in the future. However, we have to be cautious as some of these therapies are not yet found to be effective in phase III trials in humans.^164^ In the process of proving clinical efficacy of these drugs, immunomonitoring data should already be obtained in an earlier stage, whereby the immunomodulatory effects of these therapies are known before registration.

### Conclusions

Apart from improving DC therapy itself, influencing the immunosuppressive character of the TME by targeting immune cells, such as Tregs, MDSCs, or TAMs, with already registered therapies could improve response rates upon DC therapy. To accomplish this, phase III clinical trials are urgently required that investigate clinical efficacy upon DC therapy combined with other treatments and registration of DC therapy for multiple malignancies. Additionally, elucidating the underlying immunological mechanism of these synergistic effects upon combination therapy will further boost the combination of DC therapy with other therapies. A better understanding will also lead to personalized combination therapy, wherein DC therapy will be combined with other therapies based on composition of the TME, the expression of inhibitory molecules on the surface of tumor and immunosuppressive cells, and tumor mutational burden (Figure 1).

## Author Contributions

R.A.B. wrote the paper, contributed to the conception of the work and the interpretation of data, drafted the paper, and has approved the submitted version. J.G.J.V.A. and H.V. contributed to the conception of the work and the interpretation of data, drafted the paper and substantively revised it, and approved the submitted version.

## Conflicts of Interest

J.G.J.V.A. reports receiving commercial research grants from Amphera and Roche; holds ownership interest (including patents) in Amphera BV; and is a consultant/advisory board member for Amphera, Boehringer Ingelheim, Bristol-Myers Squibb, Eli-Lilly, MSD, and Roche. No potential conflicts of interest were disclosed by the other authors.

## Supplemental Information

Document S1. Supplemental Materials and Methods and Box S1

Document S2. Article plus Supplemental Information
