# Supplementary material for: Enhancing Dendritic Cell Therapy in Solid Tumors with Immunomodulating Conventional Treatment
Source: Mol Ther Oncolytics. 2019 Mar 27;13:67–81. doi: 10.1016/j.omto.2019.03.007 (PMC6475716; doi:10.1016/j.omto.2019.03.007)
Supplement: Document S1. Supplemental Materials and Methods and Box S1 [file mmc1.pdf]

OMTO, Volume *13*

Supplemental Information

Enhancing Dendritic Cell Therapy
in Solid Tumors
with Immunomodulating Conventional Treatment

Robert A. Belderbos, Joachim G.J.V. Aerts, and Heleen Vroman

Supplementary materials

Search for Medline

Medline Epub (Ovid): 2572

((((dendritic *OR TARP*) ADJ6 (vaccin* OR therap* *OR immunotherap**))).ab,ti.**)** **AND** **(**exp *Neoplasms by Site/ OR exp *Neoplasms by Histologic Type/ *OR exp *Neoplasm Metastasis/* OR (cancer* OR neoplas* OR mesothelioma* OR malignan* OR melanoma* OR carcinoma* OR glioma* *OR metasta**).ab,ti.**)** **NOT** **(**exp animals/ NOT humans/**)** **NOT** (congresses OR editorial).pt.

Box1. ICD in cancer cells

Cancer cells undergoing ICD start expressing calreticulin (CRT) on the cell surface due to stress on the endoplasmatic reticulum and excrete ATP and high mobility group box1 (HMGB1). ATP is a stimulus for the migration of DCs into the tumor, CRT stimulates phagocytosis of antigens and HGMB1 upregulates antigen presentation to $CD8^+$ T-cells. Through these processes DCs are matured and activate CTLs in the lymph node to engage cytotoxic activity on the tumor cells.[23, 120, 146] ICD can be induced by several chemotherapeutics, fractioned radiotherapy, oncolytic viruses and some targeted therapies.[1]
